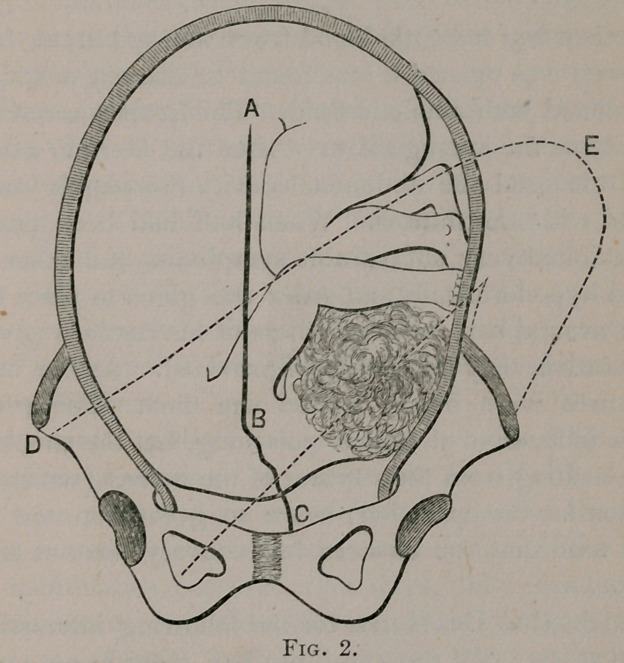# A Case of Cæsarean Section

**Published:** 1886-03

**Authors:** E. Miller

**Affiliations:** Florence, S. C.


### A CASE OF CÆSAREAN SECTION.

BY E. MILLER, M. D., OF FLORENCE, S. C.

Pelvic deformities in women being very rarely met with in my own State, as well as the other obstacles to delivery which call for the performance of gastro-hysterotomy, this operation has been one of very great rarity when compared with the statistical records of several of our States. Of 136 cases collected by Dr. Robert P. Harris, of Philadelphia, nearly one-half of which have never been published by the operators, but two belong to the credit of this State, the first being that of the King of Edisto Island, who operated in 1816, and Dr. J. Walter Hill, of Edgefield, who operated, on October 9, 1869, upon a colored woman of 32, in her second labor, the cause of difficulty arising from vaginal obstruction following her first delivery twelve years before. This labor was one of six days, and was followed by inflammation, sloughing, contraction of the vagina, and a vesico-vaginal fistula. The pelvis was believed to be normal. Although the second labor lasted fifty-six hours, the woman and her son,

"Julius Cæsar Gray," were saved; the abdominal wound healed in eleven days, and the boy is still living. Dr. Hill writes me, "and is a robust lad of sixteen, a specimen of good health, and well grown for his age. His mother is also living, has been in good health since the operation, but has never been pregnant."

Two additional cases that have never been published should be credited to South Carolina; both, however, proved fatal to mother and child. In August, 1856, Dr. T. R. Bass, after every effort to obtain the aid of a competent surgeon, as an absolute necessity operated on a woman, aged 23, white, whose coxyx and pubic bones did not seem to be more than one and a half inches apart, and it seemed impossible for the smallest child to be delivered with any appliance of art. Her condition was good, pulse normal, with some prostration from four days' hard labor. Operation occupied five minutes. Chloroform used, six white silk sutures closed the wound, passed from within out, additional support was given by adhesive strips. The child, a large, fine boy, in feeble condition, rallied and did well, but sometime after died of dysentery. The mother rallied well from the operation, took a severe chill twenty-four hours after, followed by fever which never ceased. She died on the tenth or eleventh day. I am indebted to Dr. G. W. A. McRae, of Florida (who writes from memory), for the above information.

The other case occurred in the practice of Dr. Lunney, of Darlington, about 1877, the precise date I have not been able to obtain, as no record of the case had been preserved. The patient lived near Darlington, was colored, age unknown, antero-posterior and transverse diameters of the pelvis were both too small; in labor five days. Craniotomy had been previously performed by Dr. Dargan, woman exhausted at the time of the operation, chloroform, death thirty hours after the operation. Dr. Lunney believes the mother could have been saved by an earlier resort to gastro-hysterotomy.

Although my own case had an unfortunate termination, owing largely to the low grade of health in the woman, complicated by eclampsia and a rupture of the uterus, I believe it my duty to report it, as I think all should be, for the benefit of obstetrical science. If all cases, whether successful or fatal, had been published, the work of collecting them would not have occupied the attention of Dr. Harris for sixteen years, and the medical world would have been much more generally acquainted with the causes of fatality and the requisites for success. Unfortunately for the cases operated upon, the accoucheur is seldom the operator, and the services of the latter are too often called for when it is too late to make his work a success. The high rate of fœtal death in American cases is indicative of this, and the low rate of fatality, both in mothers and children, when the operation is *timely*, shows clearly that a very early resort to the knife is a lesson that should be learned by every one engaged in the practice of obstetrics, particularly among the poor of cities. But one other case of *gastro-hysterotomy* is recorded by Dr. Harris, in which a rupture of the uterus was found to have taken place when the abdomen was opened, and was enlarged by incision for the delivery of the fœtus. This case occurred in Ohio, in 1833, and resulted in the recovery of the woman, but the child was lost: the rupture was a very slight one. This form of case must not be confounded with that of uterine laceration where the rent is sufficient for the exit of the fœtus, and in which delivery is effected by *laparotomy* without uterine incision; such cases have no title to the name *Cæsarean.*

CASE.—The subject of my operation was a deformed and dwarfed mulatto, primipara, in her twentieth year, who had been confined to bed for fourteen years, during which period she had not been able to walk; she would probably have measured about four feet or four feet two inches. She had been the subject of double coxalgia, and both joints were anchylosed, giving rise to a pelvic deformity, by which the superior strait was contracted in its conjugate to perhaps two and a half inches. Both of the lower extremities were flexed upon the body, and the right one so much so that it rested obliquely upon the abdomen and indented it by pressure. They were much emaciated, were œdematous, and broken out with small sores and a scaly eruption; there was also a deep, ragged ulcer midway between the vulva and anus, which presented the characteristic marks of syphilis. The woman lived in poverty, and weighed but sixty pounds; she also presented in her legs some evidences of having been rickety in early childhood.

When taken in labor under the care of Dr. Jarrot, of Florence, she in time was seized with convulsions, for which he gave her two grains of sulphate of morphia, which, not relieving her, he called upon me, and at my suggestion administered two more grains hypodermically, after which the convulsive movements ceased. I saw her for the first time on the morning of February 20th, 1885, and found her breathing heavily. Her abdomen was tympanitic above the uterus, and there were no uterine contractions, these having ceased suddenly, giving rise to the belief that she may have ruptured her uterus. The os uteri was found of the size of a twenty-five cent piece, and the tissues relaxed; there was but little hemorrhage. We decided, under all the difficulties of the case, to deliver by Cæsarean section. (Fig. 1.)

FIG. 1.

At the time of the operation the woman had been more or less in labor for thirty hours, and twenty-two hours had passed since she had taken any morphia. She had a feeble pulse of 118, a respiration of 20, and her extremities were cold; being unconscious, no anæsthetic was used. An incision six inches long was made in the median line, and nearer to the umbilicus than the symphysis pubis, the flexure and anchylosis of the lower extremities making this necessary. The distended intestines were with much difficulty kept back, and were punctured in several places with a hypodermic needle to evacuate the gas. The incision of the uterus led into a rupture of the lower part of the body and cervix, opening the organ to the extent of about eleven inches, six inches of which were by incision. Although laceration was believed to have taken place, it had not been postively located until cut into by the knife. After the uterus was opened the fœtus was seized by the left arm and delivered, and the cord cut

FIG. 2.

and tied. The uterus, in its contractions, now presented under the eye the curious phenomenon of inversion, the right antero-lateral portion, to which the placenta was attached, being driven through the uterine wound with the disk firmly adherent. (Fig. 2.) Although an effort was made to detach the placenta, it seemed only to become more firmly fixed as the convexity outward increased and the parts became consolidated by contraction. Finding the placenta immovable, I with my two thumbs placed on either side of the cord at its origin, and my fingers spread out over the back of the uterus to antagonize them, readily indented the protruding portion, which, being started, became restored by a spontaneous movement. The uterus now contracted normally, and the placenta was gradually separated, the mouths of the blood-vessels being closed so perfectly that it was difficult to distinguish its former site.

The uterine wound was closed by thirteen silk sutures, but a portion of the rent in the neck was not sewed up, it being inaccessible because of its position under the right thigh; after the organ had fully contracted, the laceration was not distinguishable.

The patient lost but little blood from the operation, but when the abdomen was opened it was found to contain a considerable quantity mixed with amniotic fluid. The fœtus was not weighed, but was above the average size. After the uterine wound was closed, I cleansed the abdominal cavity thoroughly and closed the wound with ten sutures. When half had been passed, the patient exhibited very unfavorable symptoms, and ether was administered hypodermically; an order was given to place bottles of hot water around her, but, regardless of instructions given prior to the operation, they had not been provided. As the last abdominal sutures were being passed she died. There were no symptoms indicative of opium poisoning, but the shock and depression resulting from the rupture of the uterus (which was the main reason for the operation) were so great that we believed from the first that the chances for recovery were as ten to one against her.

I am indebted to Dr. Harris for the following interesting facts and observations ; "Of the 136 Cæsarean cases in my note-book, but one other died during the operation, which was performed in New York city in 1860 upon a patient nearing the maturity of gestation, but not in labor, who had been for six hours in convulsions. In no other American case is there a record of uterine inversion. This may be partly accounted for in your own by the extreme length of the uterine opening. As the organ had been a long time in a quiescent state and partly emptied, any slight traction upon the cord in removing the fœtus might indent the flaccid uterine wall and favor inversion if muscular contraction was at that moment revived. I do not remember to have seen any similar accident recorded of any foreign case. Your operation increases the list of American dwarf cases to 27 and the deaths thereof to 20; twelve children were delivered alive; the number of Cæsarean operations in the United States is upon the increase, but their proportionate mortality much more than keeps pace with the growth in number. From April 7, 1884, to April 20, 1885, there were eight operations, all fatal but the last, and all of the children perished; six of the cases were reported to me by letter. Of the 136 American cases, 58 have not yet been published, and but a very small fraction ever will be by their operators."

Since January 1, 1875, there have been 32 operations in the United States, saving 8 women and 14 children. In the preceding decade, 1865–1874, there were also 32 operations, saving 8 women and 10 children. From 1855 to 1864, 27 operations, with 12 women and 11 children saved; and from 1845 to 1854, inclusive, 23 women operated upon, saving 13 of them and only 7 children. We are certainly retrograding in the measure of success, due very largely to delay; of the last 50 operations, 9 were performed early and 41 late; hence the number of deaths both of women and children.

Since my operation there have been two women subjected to the same method of dealing. I learn from Dr. Harris these cases have not been published yet. One case was under Dr. Lungren, of Toledo, a homœopathic surgeon—woman four and a half days in labor, membranes ruptured two days, child dead two days, woman exhausted, respiration 36, temperature 102 1-5, pulse 130, recovered 33. Single, Irish, primipara, 4 feet 11 inches; strumous, weight 94 pounds, date, April 19, 1885.

The other operator was Dr. Parrish, of Philadelphia, September 20, 1885. Woman white, multipara, 35, German, forty-two hours in labor, membranes ruptured thirty hours. Three febroids in lower segment of the uterus, pulse 124, much exhausted, had been under care of a midwife; operation in Philadelphia hospital. Woman died in twelve hours of exhaustion and septicuma, child dead and putrid. Uterine wound treated after Sauger's method. From January 1st, 1880, to present time, there have been in the United States twenty Cæsarean operations; of the women, five were saved; eight children were delivered alive, of which one lived only four hours and one thirty-two hours. Dr. Lungren saved two of the five women and one child.

---

How Shall the Practitioner Disinfect His Hands?—A thoroughly efficient disinfection of the physician's hands, remarks the *Therapeutic Gazette*, is more than a matter of personal cleanliness; it is an absolutely required, though often neglected, protection of his own person and the safety of his family, friends and patients. There being no dissenting voice as to the necessity of this by no means irksome precaution, the only question that can arise in this respect is, what method of disinfection insures the greatest success? The present state of bacteriology must convince even the most skeptic and conservative physician that soap and water exercise not the slightest influence over the microbial organisms, and that the true antiseptic agents have to be resorted to. Foster, of Amsterdam, made some special researches in this field (*Pharm. Centralblatte*, May 28, 1885) with the view of ascertaining the relative worth of carbolic acid, boric acid, chloride of zinc and iron. He gained the conviction that the ordinarily used two and one-half per cent. solution of carbolic acid, and even Billroth's plan to wash the hands in muriatic acid and ten per cent. phenol in glycerine, were insufficient to sterilize the hands, that is, prevent microbic growth on them. The only process that Forster found absolutely reliable was recently recommended by Koch, of Berlin, which consists in a solution of corrosive sublimate having a strength of seven to fifteen grains to two pints of distilled water. The simplicity of the manœuvre and its unquestionable prophylactic power will go far to recommend Koch's wash to the American practitioner.—*Boston Med. and Surg. Journal.*

## Figures and Tables

**Fig. 1. f1:**
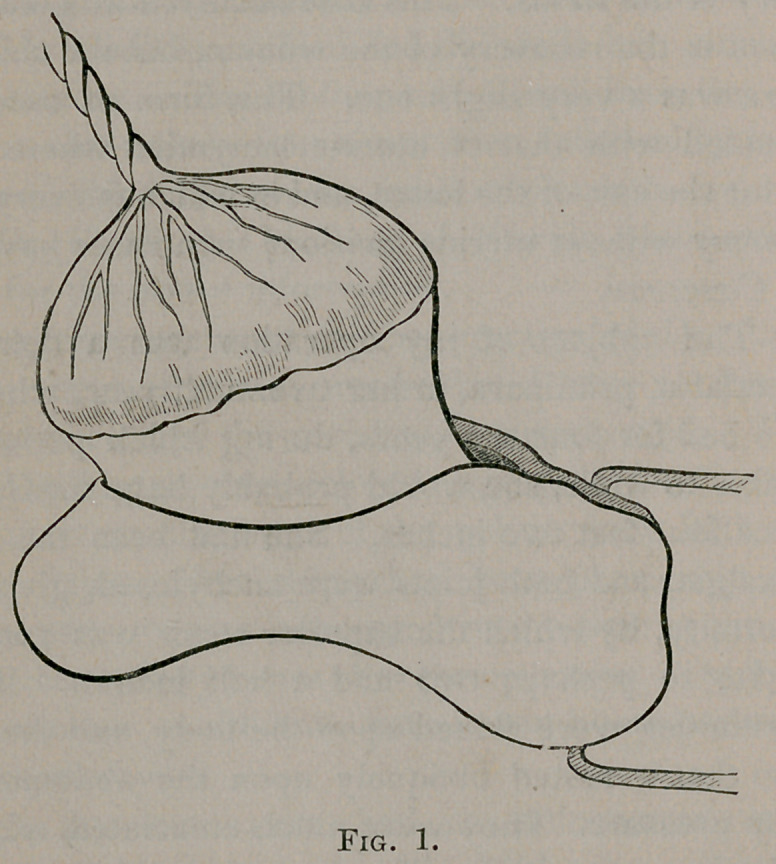


**Fig. 2. f2:**